# Psychometric properties of the Benzodiazepine Dependence Self-Report Questionnaire – Portuguese Version (BENDEP-SRQ-PV)

**DOI:** 10.1590/2237-6089-2019-0049

**Published:** 2020-11-17

**Authors:** Daniela F. Curado, Viviam V. de Barros, Emérita S. Opaleye, Ana Regina Noto

**Affiliations:** 1 Núcleo de Pesquisa em Saúde e Uso de Substâncias Departamento de Psicobiologia Universidade Federal de São Paulo São PauloSP Brazil Núcleo de Pesquisa em Saúde e Uso de Substâncias (NEPSIS), Departamento de Psicobiologia, Universidade Federal de São Paulo (UNIFESP), São Paulo, SP, Brazil

**Keywords:** Benzodiazepines, Z-drugs, hypnotics and sedatives, substance-related disorders, psychometrics

## Abstract

**Objective:**

To assess psychometric properties of the Benzodiazepine Dependence Self-Report Questionnaire – Portuguese Version (BENDEP-SRQ-PV) in a sample of Brazilian chronic hypnotic users.

**Methods:**

One hundred and seventy-nine chronic hypnotic users (benzodiazepines and Z-drugs) were recruited, attended a psychiatric evaluation, and answered the BENDEP-SRQ-PV. Factor structure, reliability, and influence of covariates (dependence diagnosis and type of drug consumed) were assessed in a structural equation modelling environment. Discrimination was assessed with receiver operating characteristic (ROC) plots and stability with the test-retest method.

**Results:**

Participants, mostly women (91.6%), aged 51 to 64 years old, had been using hypnotics for an average of 34.8 months, with a mean defined daily dose of 0.72. Psychometric analysis demonstrated construct and criterion validity, reliability, and response stability. The factor structure was maintained as originally proposed: problematic use (ω = 0.73), preoccupation (ω = 0.74), lack of compliance (ω = 0.74), and withdrawal (ω = 0.93).

**Conclusion:**

The BENDEP-SRQ-PV is an adequate measure of hypnotic dependence in the Brazilian population of chronic users. Our results support using the scale for follow-up in clinical and research applications and in correlational studies.

## Introduction

Benzodiazepines (BZD) and Z-drugs are psychotropic medications, recommended for short-term management of anxiety and insomnia,^1 , 2^ since it is recommended that treatment duration should not exceed four weeks.^3^ However, an estimated 1 in 5 patients given a first prescription become chronic users, as described by Schonnman et al. in a large-scale longitudinal study in Israel.^4^ A study investigating the period from 1993 to 2007 examined trends in prescription of hypnotics and found that while growth in use of BZDs had remained low, there had been a 30-fold increase in Z-drug use.^5^ More recent epidemiologic data (2006-2014) shows a downward trend in prescription of several hypnotics, except for clonazepam and zolpidem, which increased.^6^ There is growing evidence regarding the side effects associated with hypnotic medications. Long-term use of both high and therapeutic doses of BZDs can cause cognitive and psychomotor deficits, increasing risks of falls, fractures, traffic accidents, mortality, abuse and dependence.^3^ The same has been observed for Z-drugs, both for the impairments listed above^7^ and also for dependence,^8^ even though they were originally marketed as safer substitutes for BZDs.^1^

Only a few existing instruments were developed to measure hypnotic use and dependence. Initially, such instruments mostly measured aspects of drug withdrawal.^9 - 13^ More recently, with the acceptance of the biopsychosocial model of dependence, it has been shown that physical signs are not sufficient measures of dependence and that the psychological and social dimensions are also prevalent in the population of benzodiazepine users.^14^ Therefore, self-report instruments that also consider the psychosocial aspects of dependence,^15 - 17^ or craving^18^ were developed. Most of these questionnaires left aside withdrawal aspects and have not been validated in Brazil, except for the Severity of Dependence Scale, which is not specific to hypnotics.^19^ A study aiming to evaluate the homogeneity of the criteria for dependence from the International Classification of Diseases, 10th revision (ICD-10), and the Diagnostic and Statistical Manual of Mental Disorders, 3rd edition, Revised (DSM-III-R), demonstrated that the abstinence criteria could possibly constitute a separate dimension.^20^ Further efforts to measure the hypnotic dependence construct demonstrated that a multidimensional approach would be the most appropriate.^15^

Kan et al. developed the Benzodiazepine Dependence Self-Report Questionnaire (BENDEP-SRQ) because of the nonexistence of multidimensional instruments that reflected benzodiazepine dependence severity comprehensively and considered aspects of drug withdrawal.^17^ Creation of the scale involved a representative sample of Dutch benzodiazepine users. Further studies were conducted to evaluate the BENDEP-SRQ’s psychometric properties in various populations of BZD users, such as general practice patients, psychiatric outpatients, self-help patients, users of alcohol and other drugs, and chronic users in a discontinuation trial.^17 , 21 - 24^ Therefore, there is support for use of the the scale in both clinical and scientific applications.

Prescription of hypnotic medication follows a pattern of newly discovered drugs receiving a good press of being potentially safer than their predecessors, without their undesirable side effects. Currently, Z-drugs figure as the successors to BZDs and it has been observed that the number of prescriptions for such medicines has increased over the last few years. There are no instruments that specifically target dependence on benzodiazepines and Z-drugs available in Brazilian Portuguese. Although the BENDEP-SRQ was originally developed to assess BZD dependence, it presents questions in a neutral manner. Validation of an instrument that specifically targets hypnotic dependence, but could include Z-drugs, is relevant in the context of a need for more up to date studies assessing this construct. Therefore, the objective of this study is to evaluate the psychometric properties of the Benzodiazepine Dependence Self-Report Questionnaire - Portuguese Version (BENDEP-SRQ-PV) for the Brazilian population.

## Methods

### Study design

An observational cross-sectional study was conducted at the Drug Dependence Unit (Unidade de Dependência de Drogas [UDED]) run by the Universidade Federal de São Paulo (UNIFESP). Data were collected between July 2017 and January 2018. All procedures were submitted for approval by the university’s research ethics board (CAAE 69303817.0.0000.5505, nº 2.423.738) and written consent was obtained from all the participants.

### Participants

The sample comprised 179 chronic hypnotic users, recruited using several different means of communication. Sample size was calculated based on recommendations of a minimum of 5 participants per question on the instrument being validated.^25^ The following inclusion criteria were applied: participants over the age of 18 and literate in Brazilian Portuguese, since they needed to be able to understand the content of the BENDEP-SRQ-PV items; the criterion for chronic use was established as use of BZD or Z-drugs for at least 3 months, with a minimum frequency of once a week.

### Procedures

The author of the BENDEP-SRQ^17^ was contacted and gave permission for the validation process. The first part of the study consisted of translation and cultural adaptation of the scale, following steps proposed by Beaton et al.^26^

Potential participants were requested to contact the research staff to undergo brief phone screening covering the inclusion criteria. A psychiatric evaluation was then scheduled that was based on ICD-10 criteria for mental and behavioral disorders due to use of sedatives and hypnotics – dependence syndrome (F13.2).^27^ Presence of psychiatric symptoms, medical supervision, and usage history were also assessed.

Self-report instruments were administered after the consultation. They were administered using the RedCap platform hosted at UNIFESP^28 , 29^ or in pen-and-paper format. Completion of all questionnaires was supervised by the main investigator. As a benefit, participants were invited to participate in meditation groups (using the mindfulness-based relapse prevention [MBRP] protocol) free of charge.^30^

### Measures

The following self-report instruments were administered:

- Sociodemographic questionnaire: age, gender, monthly income, years in education, and marital status
- Characteristics of medication use: name of the medication, dosage, weekly frequency, duration of use, prescription, and medical supervision.
- Benzodiazepine Dependence Self-Report Questionnaire - Portuguese Version (BENDEP-SRQ-PV): this is the instrument undergoing validation. It comprises 20 items divided into the following domains: degree of perception of problematic use of BZD; degree of preocupation realated to obtaining the medication; lack of compliance with medical prescription; and the extent to with the patient feels troubled by commonly-reported withdrawal symptoms. Withdrawal items are only answered by participants who have previously tried to reduce/cut down their medication use. All questions are answered on a 5-point Likert scale, with options for the first 3 domains varying from “that is absolutely untrue for me” to “that is absolutely true for me”. For the withdrawal subscale, possible answers range from “none or hardly any trouble” to “a very great deal of trouble.”^17^ More material about the scale is available online at https://sites.google.com/site/bendepsrq/
- Test-retest: After an interval of 3 weeks, participants were invited to answer the BENDEP-SRQ-PV and the questions on characteristics of medication use again. The format used to complete the questionnaires was maintained constant (pen-and-paper or RedCap).

### Data analyses

All analyses were conducted with R software, version 3.6.1, using the packages lavaan^31^ and semTools^32^ . For descriptive analysis, categorical variables were expressed as frequency and percentage and continuous variables were expressed as mean and standard deviation. We conducted a comparative analysis of several theoretical models using strucutral equation modeling (SEM) with weighted least squares mean-variance (WLSMV) estimation to assess the factor structure of the BENDEP-SRQ-PV. The theoretical rationale behind the choice of the models tested was drawn from two articles: 1) Kan et al.^17^ developed the original questionnaire, dividing the BZD dependence construct into 4 domains; 2) Kan et al.^20^ analyzed the homogeneity of DSM-III-R and ICD-10 criteria for hypnotic dependence and concluded that withdrawal symptoms could reflect a separate dimension.

The models were built in several steps, as follows: Model 1 consisted of all items generating a single latent factor (hypnotic dependence); Model 2 included the domains originally described by Kan et al.,^17^ as 4 intercorrelated latent variables (problematic use, preoccupation, lack of compliance, and withdrawal); in Model 3, problematic use, preoccupation, and lack of compliance formed a second-order hierarchical factor named “psychosocial signs of dependence”. Withdrawal was maintained as a first-order factor (physical signs of dependence). These two latent variables were correlated.

Goodness-of-fit parameters were assessed to evaluate the adequacy of the models. Parameter selection and cutoff indices were based on recommendations for sample sizes smaller than 250 individuals and number of observed variables between 12 and 30.^33^ The following were presented: χ^2^ and degrees of freedom model, expecting significant values; comparative fit index (CFI) higher than or equal to 0.95; standardized root mean residual (SRMR) smaller than 0.08 and root mean square error of approximation accompanied by 90% confidence interval (RMSEA [90% CI]) smaller than 0.08.^33^ Perry et al. demonstrated that measures that are typically employed in research studies may not achieve strict fit criteria when tested in independent samples and may not be discredited.^34^ Hair et al. argue in favor of determining the superiority of one model when compared to others, as it is hard to absolutely determine, based solely on fit parameters, whether a model has good or bad fit.^33^ Therefore, the approach taken for this study was to choose the best fitting model based on parsimony (less complex models were preferred), fit parameters that are closer to cutoff indices, and model comparison. Significant increases in model fit were assessed using a χ^2^ -based likelihood ratio test.

To evaluate differences in BENDEP-SRQ scores between users of BZD or Z-drugs, we tested multiple indicators multiple causes (MIMIC) models, following the methodology proposed by Brown.^35^ The choice of the base model was based on the best fit to the data (Model 2). The covariates of interest (type of drug used) were added to this model. Significant paths between a latent variable and the covariate indicate population heterogeneity. An item was considered to present differential item functioning (DIF) when a significant path (considered when modification indices > 4) existed between the covariate and the item. The same methodology was employed to compare the mean scores of patients diagnosed as dependent to their non-dependent counterparts, by adding dependence diagnosis (ICD-10) to Model 2. Model 2 also served as the basis for calculating McDonald’s ω reliability coefficient, available in the semTools package.^32^ A cutoff point of 0.7 was considered indicative of adequate construct reliability.^33^

Latent trait values were extracted and used in further analysis. To evaluate whether scores obtained in the BENDEP-SRQ-PV were able to discriminate patients diagnosed with dependence from those who were not, we evaluated criterion validity by analysis of the area under the receiver operating characteristic (ROC) curve, using diagnosis according to ICD-10 criteria as response variable. Further evidence of reliability was obtained with the test-retest methodology, using intraclass coefficients (ICC) and defining values from 0.5 to 0.75 as moderate.^36^

## Results

### Descriptive statistics

Sociodemographic data and characteristics of hypnotic drug usage are shown in Table 1 . The 179 participants were mostly women (91.6%), with a mean age of 52.1 (±13.1) years. The majority reported a mean household income of 1 to 6 times the minimum wage (62.7%), and 59.9% reported that another family member was responsible for at least a part of the monthly income.

**Table 1:** Sociodemographic data and general characteristics of hypnotic use in the sample (n = 179).

Variable	n	%
Gender – female (missing = 0)	164	91.6
Age (missing = 0)		
18 to 35 years	24	13.4
36 to 51 years	47	26.3
51 to 64 years	78	43.6
Older than 65 years	30	16.8
Marital status (missing = 0)		
Single	52	29.0
Married	76	42.5
Separated/divorced	40	22.4
Widowed	11	6.1
Schooling (missing = 0)		
Incomplete to complete junior high	19	10.6
Incomplete to complete high school	38	21.3
Incomplete college to complete college	78	43.5
Postgraduate studies	44	24.6
Health service prescribing the medication (missing =1)		
Basic health care unit	31	17.4
Private doctor’s office	112	62.9
Hospital	12	6.8
Not taken under medical supervision	23	12.9
Number of hypnotic medications in current use (missing = 1)		
1	167	93.3
2	11	6.1
3	1	0.6
Current hypnotic used* (dosage/day) (missing = 0)		
Alprazolam (0.2-2 mg)	26	14.5
Bromazepam (3-6 mg)	3	1.7
Clonazepam (0.1-6 mg)	61	34.1
Diazepam (5-20 mg)	4	2.2
Lorazepam (0.5-4 mg)	8	4.4
Zolpidem (1.25-35 mg)	84	46.9
Others^†^	6	3.3

* Percentages do not add up to 100% because the answers to this question are not mutually exclusive.

^†^ Medications in the “others” category were: estazolam, clobazam and chlordiazepoxide, cloxazolam, flurazepam, and zopiclone (1 user of each).

On average, participants began using any type of hypnotic (BZD or Z-drug) at 41.9 years of age (±14.8). The mean duration of current medication use was 34.8 months (±49.8), the minimum duration was 3 months and the maximum was 300 months. Seventy-five (41.9%) participants reported some duration of interrupted use (meaning usage for a period in the past and then withdrawal or consumption of another type of medication that was replaced for the current one). The minimum duration of interrupted use was 3 weeks and the maximum was 480 months, with an average of 72.2 (±107.7) months. Mean defined daily dose was 0.72 (±0.65). Twenty-three participants (12.9%) reported that they did not have a medical prescription for the hypnotic and most of these acquired the medication through a friend or relation who was a doctor (10.6%).

### Psychometric properties of the BENDEP-SRQ-PV

Seven out of 179 participants had missing values for at least one of the 20 items and their data were therefore excluded from further analysis.^33^ For participants who did not respond to the withdrawal questions because they had never tried to reduce their medication use, these answers were replaced with zeroes. This procedure was used so that analyses were not run using only data from those who had tried to reduce their medication use (82.1% of our sample).

### Factor analysis

**Figure 1:**
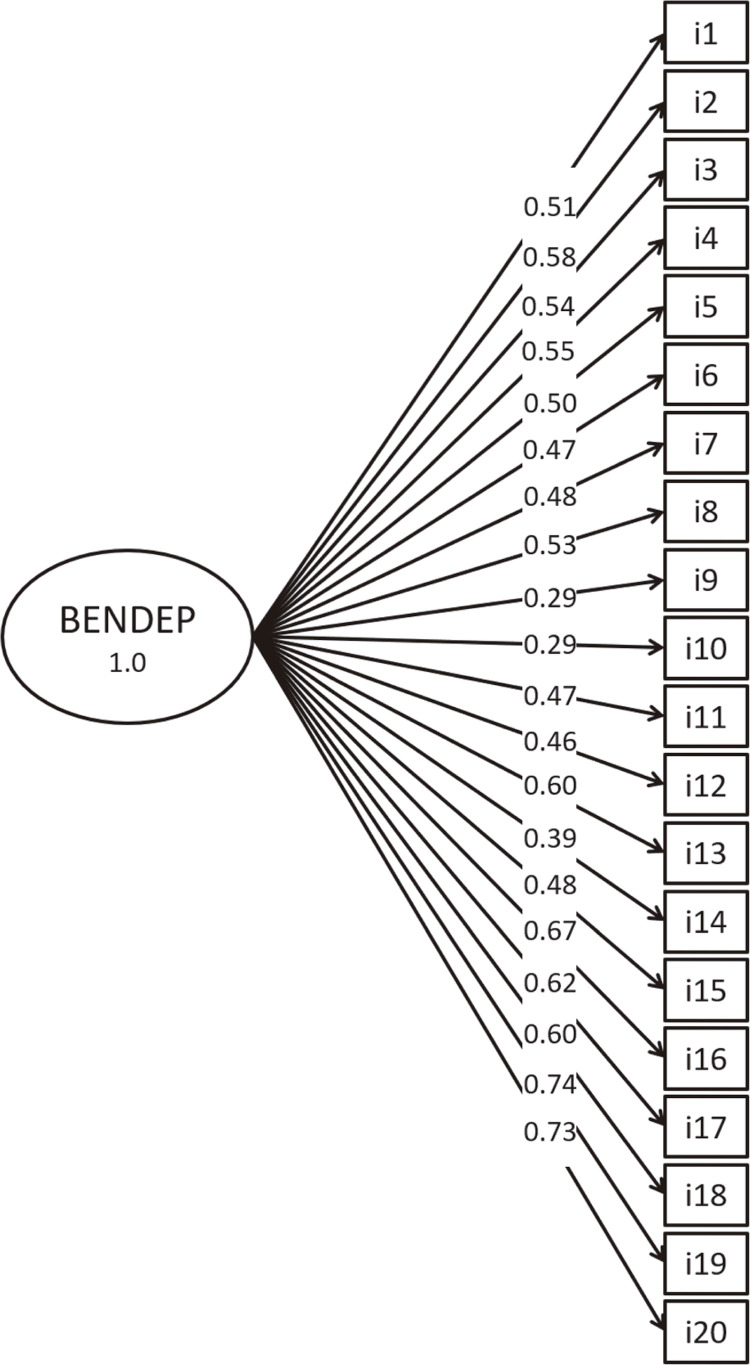
Model 1, a unidimensional model in which all of the BENDEP-SRQ-PV items load onto a single hypnotic dependence factor (BENDEP). Items are numbered as on the questionnaire and represented in rectangles, the latent variable is illustrated by the oval shape, and the standardized factor loadings are shown along the single-headed arrows. BENDEP-SRQ-PV = Benzodiazepine Dependence Self-Report Questionnaire – Portuguese Version.

**Figure 2:**
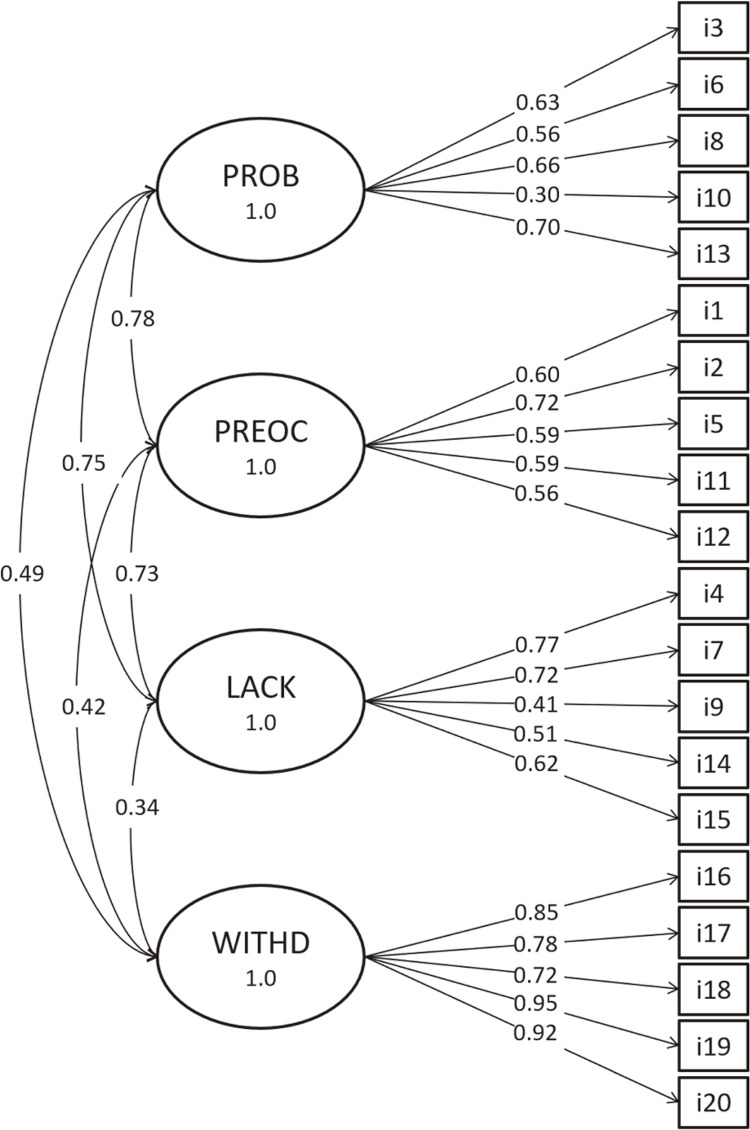
Model 2, in which all 20 items, represented in rectangles, load onto the specific factors defined by Kan et al. Items are numbered as on the questionnaire and represented in rectangles and the latent variable are illustrated by the oval shapes. Correlations between latent factors are represented along the curved double-headed arrows; standardized factor loadings are shown along the single-headed arrows. LACK = lack of compliance; PREOC = preoccupation; PROB = problematic use; WITHD = withdrawal.

**Figure 3:**
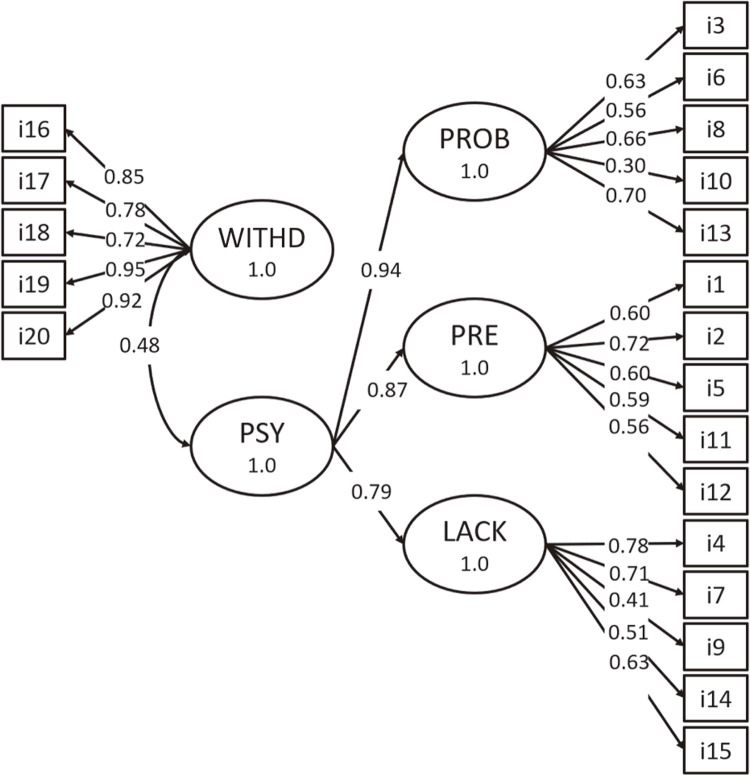
Model 3, based on work by Kan et al.,20 suggesting that withdrawal (WITHD) symptoms could constitute a separate dimension of hypnotic dependence. Problematic use (PROB), preoccupation (PRE), and lack of compliance (LACK) are grouped in a second-order hierarchical factor named psychosocial signs of dependence (PSY). Withdrawal was maintained as a first-order factor (physical signs of dependence). Items are numbered as on the questionnaire and represented in rectangles and the latent variables are illustrated by the oval shapes. Correlations between latent factors are represented along the curved double-headed arrows; standardized factor loadings are shown along the single-headed arrows.

Model 1, is a unidimensional model in which all items load onto a single latent factor for hypnotic dependence. It was a poor fit to the data: χ^2^ (170) = 552.652, RMSEA (90% CI) = 0.115 [0.104; 0.125], CFI = 0.559, SRMR = 0.128 ( Figure 1 ). Model 2, in which items loaded onto the four factors proposed by Kan et al., was a good fit to the data χ^2^ (164) = 226.46, RMSEA (90% CI) = 0.047 [0.031; 0.062], CFI = 0.928, SRMR = 0.069 ( Figure 2 ). In Model 3, the subscales problematic use, preoccupation, and lack of compliance formed a second order hierarchical factor, named psychosocial aspects of dependence, that was correlated to the first order withdrawal factor. This model also presented adequate fit to the data: χ^2^ (164) = 225.55, RMSEA (90% CI) = 0.046 [0.029; 0.060], CFI = 0.931, SRMR = 0.070 ( Figure 3 ). Since Model 3 was not a significant improvement over Model 2 (Δχ^2^ = 2.55, p = 0.281), we adopted Model 2 as the basis for subsequent analyses.

Under Model 2, all latent variables were significantly correlated, with values ranging from 0.78 (problematic use and preoccupation) to 0.34 (withdrawal and lack of compliance). Reliability coefficients were as follows:

$$\omega_{\mathrm{problematic}\;\mathrm{use}}\;=\;0.73,\;\omega_{\mathrm{preoccupation}}\;=\;0.74,\;\omega_{\mathrm{lack}\;\mathrm{of}\;\mathrm{compliance}}\;=\;0.74,\;\omega_{\mathrm{withdrawal}}\;=\;0.93.$$

Differences in BENDEP-SRQ PV scores between users of BZD or Z-drugs was tested by adding the covariate “drug type” to Model 2. We found effects of drug type on problematic use (β = 0.28, p = 0.002), preoccupation (β = 0.20, p=0.033), and lack of compliance (β = 0.23, p = 0.005), in all cases, BZD users tended to score higher. Addition of the covariate ICD-10 dependence diagnosis (dependent n = 130; non-dependent n = 45) yielded significant paths for problematic use (β = 0.17, p = 0.025), preoccupation (β = 0.23, p = 0.018), and lack of compliance (β = 0.19, p = 0.015). Patients who had a dependence diagnosis tended to score higher. No evidences of DIF were found for the items.

### Criterion validity and reliability

ROC analysis was used to assess whether the BENDEP-SRQ-PV can be used to discriminate between those who are dependent and those who are not. Latent traces were extracted from Model 2 and, for each domain, using an ICD-10 diagnosis of dependence as criterion, the following values for area under the curve (AUC) were encountered: problematic use (AUC = 0.63), preoccupation (AUC = 0.63), lack of compliance (AUC = 0.64), and withdrawal (AUC = 0.56). As for test-retest, 51 participants completed the characterization of hypnotic use and the BENDEP-SRQ-PV a second time after a 3-week interval. Model 2 was adjusted using these new answers and new latent trait values were computed. Intraclass correlations between the values obtained in the first and second administrations of the instrument are as follows: problematic use (ICC = 0.76), preoccupation (ICC = 0.77), lack of compliance (ICC = 0. 69), and withdrawal (ICC = 0.56).

## Discussion

Our results demonstrated that the BENDEP-SRQ-PV is an adequate measure of hypnotic dependence in the Brazilian population, both for users of BZD and for users of Z-drugs. Evaluation of the scale’s psychometric properties yielded satisfactory indices of validity and reliability. The analysis demonstrated adequate construct validity, suggesting that hypnotic dependence, measured with the BENDEP-SRQ-PV behaves as originally proposed, with four independent but correlated domains: problematic use, preoccupation, lack of compliance, and withdrawal.^17^

Patients diagnosed as dependent had significantly higher mean scores than non-dependent patients in the BENDEP-SRQ-PV domains of problematic use, preoccupation, and lack of compliance. Psychological research scales facilitate the diagnostic process, but do not function as dignostic instruments. That ROC analysis did not demonstrate sufficient sensitivity or specificity to indicate that the BENDEP-SRQ-PV could function as a diagnostic instrument confirms this. The recommended uses of this scale are for follow-up in research and clinical practice, through repeated applications, and for establishing relationships with other empirically measured constructs. These applications are in line with what was originally proposed.^17^

Our sample, mainly composed of women, aged between 51 and 64 years, is similar to what is described in the literature on people with insomnia (women, incidence increasing with age, especially post menopause),^37^ users of benzodiazepines and Z-drugs.^4^ In a study assessing the prevalence of BZD use in the Brazilian population, women aged 40+, separated/divorced, and with higher education and income had higher lifetime use prevalence rates.^38^ Except for marital status, this profile is compatible with what was observed in the current study. The long duration of use, large percentage of participants reporting medical follow-up, and consumption of low daily doses characterizes our sample as low-dose dependent.^3^

Although originally marketed as safer alternatives to BZDs, evidence regarding the potential for dependence of Z-drugs is mounting.^8^ In our study, mean scores obtained in the domains of problematic use, preoccupation, and lack of compliance were higher among BZD users than among users of Z-drugs. These results suggest milder cases of dependence among Z-drugs users, although previous studies did not find evidence pertaining to security, effectiveness, or cost-benefit ratio that favor one class over the other.^39 , 40^ These differences could be better understood by examining other factors, e.g., longer treatments are associated with a drop off in compliance with the therapeutic regimen.^41 , 42^ Considering that BZDs were introduced onto the market in the 1960s and Z-drugs were introduced in the 1990s.^1 , 43^ BZD users are more likely to present an increased duration of treatment, therefore, the increased lack of compliance could be due to treatment duration and not necessarily to the type of drug consumed.

The problematic use subscale measures the degree of awareness respondents have of their hypnotic use, which is essential in a population of users who often do not recognize their drug dependence,^44^ but can recognize individual behaviors presented in the form of items, illustrating the importance of adapting self-report instruments to assess hypnotic dependence. In a study assessing risk factors associated with benzodiazepine dependence, especially psychopathological risk factors, depression was found to partly predict problematic use, along with other sociodemographic variables such as younger age and lower educational level.^45^ Manthey et al. found associations with insomnia severity and more frequent contact with the prescriber.^46^ This domain was also associated with prolonged reaction times in an attentional task.^47^ In a discontinuation trial using melatonin, even though 71% of patients who reported a low degree of awareness of problematic use were unable to quit the medication, no significant effects were found comparing them with those who were able to quit or those with high awareness.^48^

The items and the theoretical rationale behind the preoccupation subscale reflect recently reviewed behavioral aspects of low-dose BZD dependence, such as anxiety or craving between doses and users carrying tablets with them or taking an extra dose of medication to avoid problems.^2^ Increased preoccupation scores are associated with craving for BZDs.^49^ In the above-cited discontinuation trial, individuals who successfully tapered off their medications, compared to those who did not, had significantly larger decreases in their scores for preoccupation and problematic use.^21^ Dimensions of anxiety and agoraphobia have been found to predict preoccupation scores^45^ ; other associations include anxiety, use of antidepressants, alcohol dependence and higher daily doses.^46^ The scale was recently used in a study aiming to compare the clinical presentation of long-term use of different types of BZDs. The only facet that differed between the substances evaluated was preoccupation, which was higher among lorazepam users.^50^

The lack of compliance domain is especially important because it reflects the medical aspect of dependence on hypnotics, which differentiates it from dependence on other substances. Hypnotic users often represent themselves as responsible poeple who strictly follow medical instructions. Reports acknowledging dependence or use outside the boundaries of medical prescription were loaded with a negative moral burden.^51^ Hostility has been found to predict scores in this particular domain^45^ ; other associations were found with higher age, unemployment, alcohol dependence, use of antidepressants and insomnia.^46^ When assessing the ability of each BENDEP-SRQ subscale to predict successful tapering after a BZD discontinuation trial, lack of compliance and preoccupation had the highest independent predictive values.^21^ Another discontinuation trial evaluating long-term abstinence found that lack of compliance significantly predicted success.^52^

The items on the withdrawal subscale reflect symptoms reported as barriers that prevent users from reducing or cutting down their medication, which is also a means through which they report perceiving dependence.^44^ The inability of this domain to discriminate those who are dependent from those who are not dependent is in accordance with the facts that hypnotic dependence is a biopsychosocial condition and abstinence is not its only good measure of discrimination, which might even constitute a separate dimension.^20^ No differences were found in withdrawal scores when comparing dependent and non-dependent individuals. Although this result does not devalue the domain’s importance, it highlights other relevant components of hypnotic dependence, not necessarily linked to a physical aspect, but focusing on observation of behaviors related to hypnotic consumption. In a two-part intervention, those who did not succeed in discontinuing BZD use of their own accord, after receiving a letter from their general practitioner with advice to attempt gradual reduction, and who were then required to participate in a second intervention (a discontinuation trial), had higher scores in the domains of problematic use, preoccupation, and withdrawal.^53^ Psychopathological risk factors that predicted this domain were anxiety and insufficient thinking and acting.^45^

This study’s strengths are founded on the relevance of measuring the construct of hypnotic dependence in both clinical practice and research settings.^15^ The BENDEP-SRQ is a multidimensional instrument that encompasses the biopsychosocial aspects of dependence. The content of its four domains remains current, reflecting behavioral aspects of low-dose dependence on hypnotics.^2^ This is the first study that has evaluated the psychometric properties of a scale to measure hypnotic dependence in Brazilian residents, which is relevant considering that the indiscriminate use of anxiolytics and hypnotics is considered a public health problem,^5^ and its use could further improve the methodological quality of future studies assessing the construct in question. Since the scale was originally intended specifically for assessing dependence on BZD, the results of this study demonstrate that it is also applicabile to Z-drugs.

Limitations must be noted. The sample size was estimated based on recommendations that only consider the number of items in the instrument being validated and was smaller than is desirable for conducting analyses within a SEM framework (e.g., we were unable to assess model invariance across time, because only 51 individuals answered the BENDEP-SRQ-PV a second time). Dependence was not diagnosed using structured interviews. Selection bias may have occurred because of the recruitment method, which may selectively attract people who are more motivated to stop the use of hypnotics and more aware of their problematic medication use. The population is, therefore, very homogeneous, composed mainly of those classified as low-dose dependent,^3^ which may hinder generalization of the results to other types of hypnotic users. Although women are prescribed hypnotics twice as often,^54^ men more frequently misuse such medications.^55^ Our sample was composed almost entirely of women (91.6%), which might also represent a limitation with regards to the capacity for generalizing our findings.

Future studies could cross-validate the BENDEP-SRQ-PV for other groups of hypnotic users, e.g. those on alcohol withdrawal treatment or high-dose dependent users. The longitudinal applicability of the scale could also be tested, in clinical trials aiming to reduce or cut down medication use, both to assess the scale’s sensitivity for detecting changes over the course of treatment and also as a tool for prediction of successful withdrawal. Considering the neutral presentation of the questions, the scale’s applicability to other pharmaceuticals used in psychiatric treatment could also be tested, since it has already been used for antidepressants.^56^ The development and validation studies used item response theory and future studies could also use this approach, testing the fit of more complex models (such as multidimensional graded response or partial credit models), since this methodology requires larger samples.

## Conclusion

Our data provide evidence of the factor structure and feasibility of the BENDEP-SRQ-PV for measuring hypnotic dependence among Brazilian chronic users of hypnotics, both benzodiazepines and Z-drugs, with good psychometric properties. The sample analysed was mostly made up of low-dose dependent users and so the results of those analyses are more applicable to low-dose dependent users. Recommended uses of this scale are mostly for follow-up in research and clinical practice or to establish relationships between hypnotic dependence and other empirical constructs.
